# Evaluation of FRP Bars under Compression and Their Performance in RC Columns

**DOI:** 10.3390/ma13204541

**Published:** 2020-10-13

**Authors:** Laith AlNajmi, Farid Abed

**Affiliations:** Department of Civil Engineering, American University of Sharjah, Sharjah 26666, UAE; b00077450@aus.edu

**Keywords:** Basalt Fiber-reinforced Polymer (BFRP), Glass Fiber-reinforced Polymer (GFRP), finite element, columns, compression

## Abstract

The behavior of fiber-reinforced polymer (FRP) bars under compression is not fully understood yet due to the limited research in this area. However, the long-term durability, weathering resistance, and exceptional mechanical properties of FRP bars justify the need for their use in compression members. The main objectives of this study are to evaluate the mechanical properties of glass FRP (GFRP) and basalt FRP (BFRP) bars under compression and examine their performances as main longitudinal reinforcements in reinforced concrete (RC) columns. In the first part of this research, a series of static compression tests were conducted on GFRP and BFRP bars of different diameters. The second part of this research numerically investigated the behavior of FRP-RC columns under concentric and eccentric loading using the mechanical properties of the FRP bars obtained experimentally. Nonlinear finite element models were developed to simulate the compressive behavior of the concrete columns reinforced with GFRP and BFRP bars. The FE models were verified with the experimental results conducted previously. The verified FE models are then utilized to conduct a parametric analysis considering two different column geometries and cross-sections, five reinforcement ratios, two concrete compressive strengths, three types of ties materials, and several loading eccentricities to develop a set of interaction diagrams that may provide valuable data for design purposes. The results indicated that the FRP bars could have a significant contribution to the overall capacity of FRP-RC columns by up to 35% of the total force at failure, depending on the reinforcement ratio. The performance of both the GFRP- and BFRP-RC columns was almost similar in terms of capacity, deflection, and bar strength contribution.

## 1. Introduction

In recent years, fiber-reinforced polymer (FRP) bars have been used to reinforce concrete members in tension, while their contribution in compression has been neglected due to insufficient research. The main advantages of FRP bars in comparison with steel are their very light density, larger strength, and most importantly that they do not corrode even in harsh environments. The mechanical and physical properties of FRPs are controlled by their micro-structural configuration and the properties of their constituents. FRP composites are ideal for structural applications where high strength-to-weight and stiffness-to-weight ratios are required [1]. However, the applications of advanced composite materials in civil engineering have been evolving slowly, primarily due to economic reasons. This class of materials has been extensively studied and used in the structural and aerospace engineering fields, such as aircraft construction [2]. While FRP materials can support tensile stresses, there are numerous issues surrounding the use of FRP in compression [3].

Many studies had also been conducted to evaluate the durability [4,5,6,7,8], flexural [9,10,11], and shear [12,13,14,15,16,17] performances of concrete beams reinforced with carbon (CFRP), glass (GFRP), and basalt (BFRP) FRP types of bars. Other studies investigated the effect of high temperatures on the performance of FRP bars [18]. Hybrid reinforcements of steel and FRP bars were also examined for slender beams under flexure [19,20]. However, limited research has been conducted on investigating the compressive response of FRP bars [21].

For the research that investigated the behavior of FRP bars under compression, different setups have been made, with varying ways of measurement, methods of fixing the ends, as well as strain rates [22,23,24,25,26,27]. Plevkov et al. [22] examined the behavior of GFRP and CFRP bars of 10 mm in diameter and 50 mm in length under compression. The modulus of elasticity was found to be 41 GPa for the GFRP bars, which is 67% of that in tension, and 105 GPa for the CFRP bars, which is 73% of that in tension. A study by Khan et al. [26] examined the compressive performance of CFRP bars using a simplified ASTM D695-10 [28] compression test method for rigid plastics. The modulus of elasticity in compression for the CFRP bars was 17% times greater than that of the GFRP bars, while the modulus of elasticity in compression of the GFRP bars (42.0 GPa) obtained was almost identical to the value (42.5 GPa) reported by Deitz et al. [25]. Recent studies have investigated the performance of concrete columns reinforced with FRP bars both numerically and experimentally [21,29,30,31]. The main outcome of these studies was to investigate the contribution of the FRP bars to the load-carrying capacities of RC columns as compared to steel bars. It was shown that such a contribution to the GRP bars was less than that of steel. However, the contribution of CFRP bars to the load-carrying capacity of FRP-reinforced concrete (RC) columns was the same or higher than that of steel bars.

Although international codes have recently started to permit the use of FRP bars in compression members, the lack of research in this area results in an incomplete understanding of the FRP bars’ behavior under compression. Therefore, the first objective of this paper was to provide experimental data on the compressive performance of different sizes of GFRP and BFRP bars. The experimental results were then utilized to numerically investigate the axial performance of concrete columns reinforced with these types and sizes of FRP bars (FRP-RC columns). Nonlinear finite element models were developed to simulate the axial performance of the FRP-RC columns and were validated using experimental tests conducted previously by the authors. The validated models were then used to perform a parametric analysis, considering several column geometries and cross-sections, reinforcement ratios, ties materials, concrete strengths, and loading eccentricities. The FE results were presented and discussed in terms of load vs. displacement curves, interaction diagrams, and ductility indices.

## 2. Experimental Evaluation of BFRP and GFRP Bars under Compression

**Figure 1:**
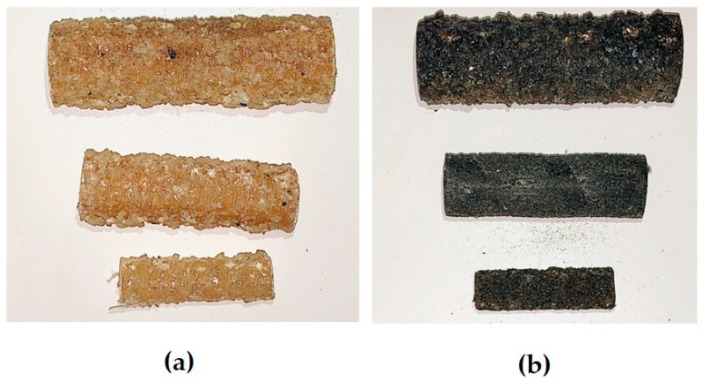
Samples of (**a**) the glass fiber-reinforced polymer (GFRP) and (**b**) basalt fiber-reinforced polymer (BFRP) bars used in this study.

In this section, the compressive properties of the GFRP and BFRP bars are experimentally investigated. Commercially produced Φ 8, Φ 12, and Φ 16 GFRP and BFRP bars were selected for this study, as shown in Figure 1.

The GFRP and BFRP bars were produced by Galen, a Russian company based in the city of Cheboksary. These GFRP and BFRP bars were manufactured by pultrusion, in which the fibers (glass or basalt) are impregnated with a polymer binder, and then run through the system drain bushing.

The tensile properties of these bars listed in Table 1 were obtained by the authors in previous work [10,27].

**Figure 2:**
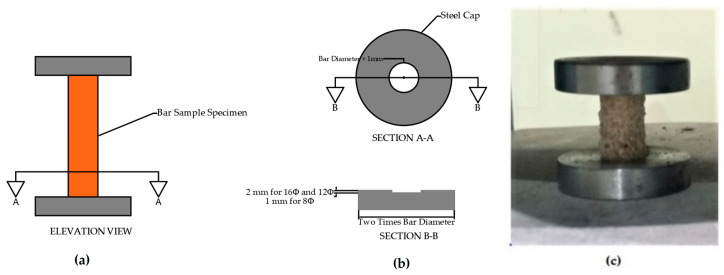
(**a**) Schematic drawing of the apparatus used for the compression tests of the GFRP and BFRP bars: (**b**) cross sections; (**c**) actual compression testing apparatus.

Several compression test setups were examined, and the test arrangement shown in Figure 2 was chosen.

It was difficult to obtain a perfectly flat end perpendicular to the loading axis with the equipment available. Restraining the ends of the specimens with a recess is illustrated in the test apparatus shown in Figure 2. It was intended to reduce the effect of the specimen tilting, which would have an effect on the test results.

**Figure 3:**
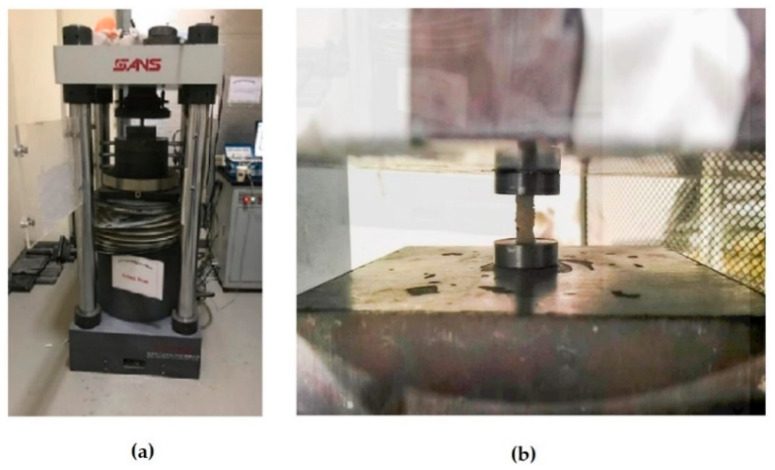
(**a**) Compression testing machine; (**b**) sample of the tested specimen.

The FRP bars were tested using a universal testing machine (UTM) with a capacity of 3000 kN under compression, as shown in Figure 3a.

The compression tests were conducted at a rate of 0.25–0.5 MPa/s. The length of each FRP bar specimen was two times the diameter. Slightly oversized holes in the ends of the testing apparatus allowed some rotation at the ends of the specimens, thereby reducing the moments applied by the apparatus while still providing some end restraint, as shown in Figure 3b.

Five bar specimens of each size were tested, and the average compressive strength and their standard deviations were reported, as shown in Table 2 and Table 3 for the GFRP and BFRP bars, respectively.

In general, the variation in the compressive strength results between the five specimens were reasonable for all sizes. However, and unlike their tensile strengths, the compressive strengths of both the GFRP and BFRP reported lower values at smaller sizes. In particular, the compressive of the 8 mm GFRP and BFRP bars were reduced by 45% and 12% as compared to 16 mm GFRP and BFRP bars, respectively. On the other hand, the compressive strengths of the BFRP bars were in the range of 35–41% of their tensile strengths. For the case of the GFRP bars, the compressive strengths of the 8, 12, and 16 mm bars were about 32, 51, and 64% of their tensile strengths.

**Figure 4:**
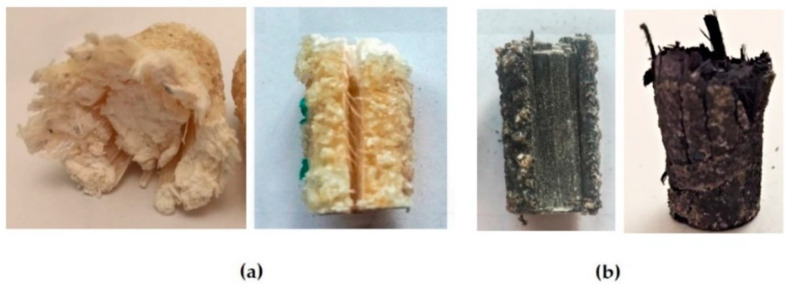
Experimental failure modes for the selected (**a**) GFRP and (**b**) BFRP bar specimens.

One distinct failure mode was observed during the tests. The failure mode was a crushing failure in which the glass and basalt fibers separated from the resin matrix, as shown in Figure 4. 

## 3. Evaluation of RC Columns Reinforced with GFRP and BFRP Bars

In this section, the axial behavior of the rectangular concrete columns reinforced with GFRP and BFRP bars is numerically investigated. Nonlinear finite element (FE) models were developed to predict the axial behavior of the reinforced concrete (RC) columns under concentric and eccentric loading. Different parameters, such as the longitudinal reinforcement ratios, different cross sections, and transverse reinforcement material for the RC columns, were considered. The compressive and tensile properties of the GFRP and BFRP bars investigated in the previous section (Table 1, Table 2 and Table 3) were utilized in developing the FE model and in conducting the parametric analysis. 

### 3.1. Finite Element Modeling

This section presents the development and verification of the FE models for concrete columns reinforced with GFRP bars (GFRP-RC columns) and BFRP bars (GFRP-RC columns). The commercial software package ABAQUS was used to create the nonlinear finite element models in which the axial behavior of the GFRP- and BFRP-RC columns were accurately simulated. The FE model verification was also performed using a set of experimental tests conducted previously by ElMesalami [32] on similar columns. 

#### 3.1.1. Materials Properties

**Figure 5:**
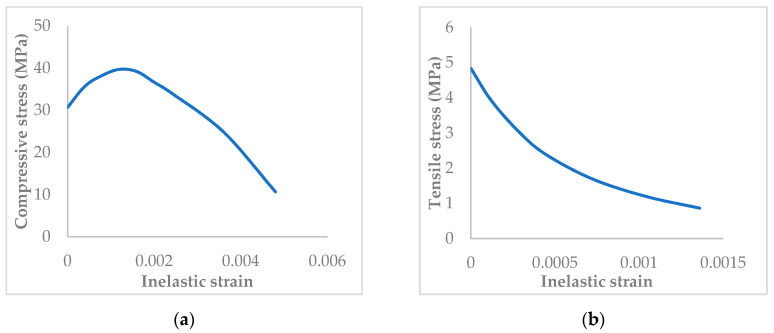
(**a**) Inelastic compressive and (**b**) tensile behaviors of the concrete used in the FE model.

The material used in the FE models included GFRP, BRFP, steel, and concrete. The concrete material was defined in the elastic zone through the elastic modulus and Poisson’s ratio while the inelastic behavior is defined using the concrete damage plasticity (CDP) model. Utilizing the CDP approach allows defining both the compressive and tensile properties of the concrete material in the FE analysis. Figure 5 illustrates the compressive and tensile properties for concrete used in the present FE analysis.

The plasticity parameters for the CDP model contains the dilation angle of 36, eccentricity of 0.1, an fb0/fc0 ratio of 1.16, parameter K of 0.667, and viscosity parameter of 1.0 × 10^−5^. The GFRP and BRFP materials are defined using the elastic modulus and ultimate compressive and tensile strengths listed in Table 1, Table 2 and Table 3. The plastic behavior for the steel ties is defined using the yield strength for Grade 60 steel reinforcement (420 MPa). 

#### 3.1.2. FE Model Geometry

**Figure 6:**
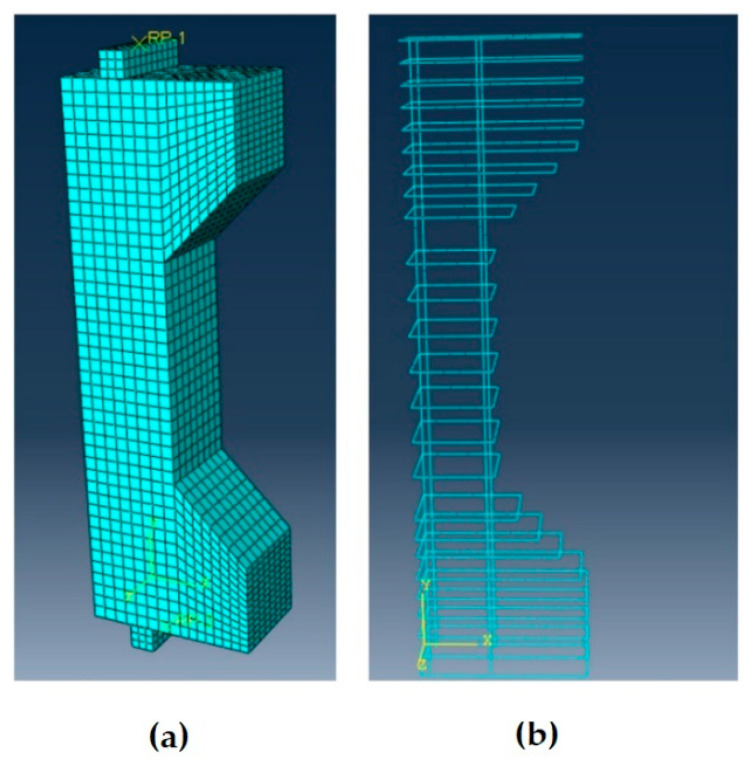
(**a**) Mesh for the square cross section of 180 mm × 180 mm × 1100 mm; (**b**) geometry of the cage.

The concrete column was modeled as a homogenous three-dimensional solid section using eight-node linear brick elements with reduced integration whereas the longitudinal and transverse reinforcements were modeled using deformable truss elements, which only carries axial load during bending. The transverse reinforcements were defined with cross-sectional areas of 78.5 mm^2^ and designed such that they were surrounded by a 27.5 mm concrete cover. Figure 6 shows the full model and the reinforcements along with the chosen mesh for the square cross-section of 180 mm × 180 mm × 1100 mm.

To simulate the interaction between the reinforcement and the concrete, the ABAQUS built-in constraint “embedment” was used. This constraint restricts the nodes of the reinforcement to the corresponding degrees of freedom of the host domain. As shown in Figure 6a, rigid plates were added to the model to ensure the uniformity of the load applied on the top and bottom surfaces.

Moreover, normal and tangential surface-to-surface contact defined the interaction between the rigid plates and concrete surfaces using the penalty contact approach. Boundary conditions and displacement were assigned on the plates through reference points defined on the center of each rigid plate.

**Figure 7:**
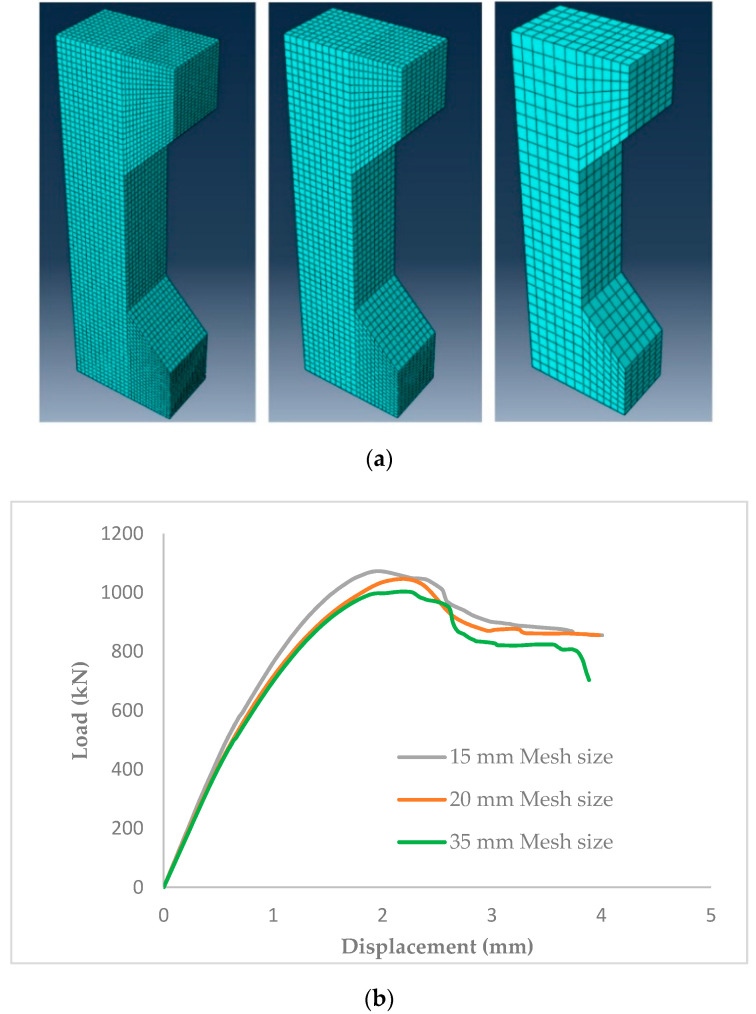
Mesh sensitivity analysis: (**a**) mesh configurations and (**b**) load vs. displacement results for the different element sizes.

A mesh sensitivity analysis was also conducted to select the appropriate mesh size that provide results accuracy with less computational cost. The model with a mesh size of 20 mm was considered throughout the analysis. The G16-0 model by ElMessalami [32] was chosen to perform a mesh sensitivity analysis, in which it was found that reducing the mesh size further will not affect the results, as shown in Figure 7.

#### 3.1.3. FE Model Verification

The experimental program conducted by ElMessalami [32] was partially to verify the FE modeling of rectangular concrete columns reinforced with GFRP and BFRP bars. The experimental program consisted of twenty-two reinforced concrete columns tested under monotonically increasing pure axial load. All columns were cast with normal-weight, ready-mixed concrete with an average compressive strength of 34.4 MPa. The verification results of only three columns were presented in this paper since the experimental results are currently submitted for publications.

Table 4 presents the details and axial capacity results for three selected column specimens.

**Figure 8:**
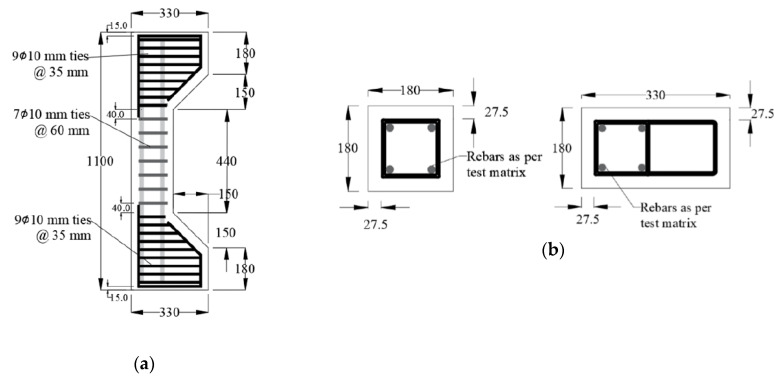
Experimental columns and cross-sections (in mm) by ElMessalami [32]: (**a**) Column dimensional details; (**b**) Column Cross Section.

The column labels represent the reinforcement type and quantity, where the first letter refers to the longitudinal reinforcement type (B = basalt, G = glass, and S = steel). The first number after the letter refers to the diameter of the longitudinal reinforcement (16 or 20 mm) and the second number refers to the eccentricity value (0, 40, or 80 mm). Figure 8 shows the geometry and cross-sections detailing of the test specimens that were used in the FE model verification.

**Figure 9:**
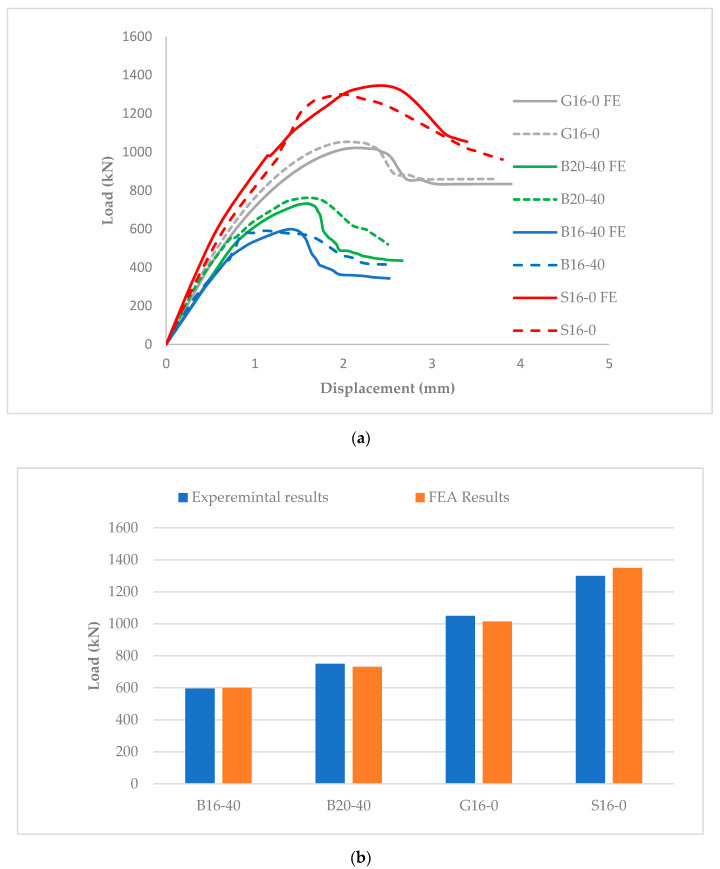
Finite element (FE) model verifications of the experiments: (**a**) load vs. displacement; (**b**) compressive strengths.

Figure 9 shows the FE verification results as compared to the experimental data for the three selected GFRP- and BFRP-RC columns.

The comparisons of the load vs. displacement curves (Figure 9a) and the ultimate compressive strengths (Figure 9b) between the FE model predictions and experiments were generally very good and within the approximate errors of 5%. Thus, the validated FE model was later used to conduct the FE parametric analysis for an extending list of columns, as discussed next.

## 4. Parametric Analysis: Performance of the GFRP- and BFRP-RC Columns

**Figure 10:**
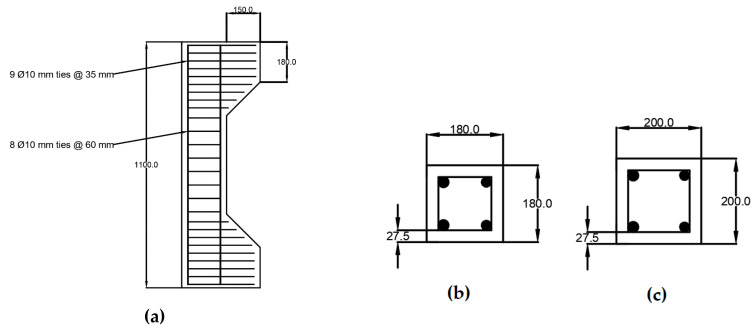
Rectangular FRP-RC column details: (**a**) column dimension; (**b**) cross section of the 180 mm × 180 mm column; (**c**) cross section of the 200 mm × 200 mm column.

**Figure 11:**
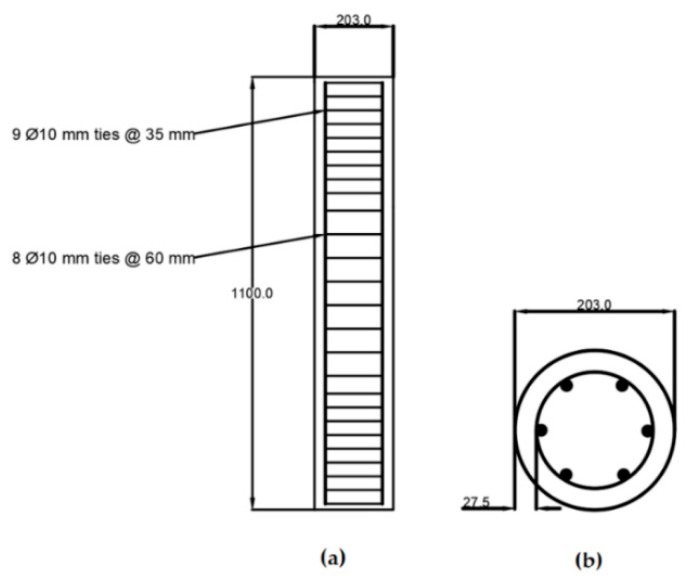
Circular FRP-RC column details: (**a**) column dimension in mm; (**b**) cross section in mm.

A parametric study was conducted to further investigate the response of the GFRP and BFRP bars in RC columns by considering different reinforcement ratios, column shapes and dimensions, concrete compressive strengths, and stirrups types. The effect of changing these parameters on the overall behavior of the RC columns is also presented and discussed. Two different column geometries with rectangular and circular cross-sections were investigated, as shown in Figure 10 and Figure 11, respectively.

The columns in the FE parametric study were divided into ten groups. Each group consisted of a total of 45 short RC columns, including a total of nine load eccentricities with five reinforcement ratios of 1%, 2%, 4%, 6%, and 8% for each load eccentricity, as listed in Table 5.

Each column was labeled with a unique ID in each group. The first letter indicates the column cross-section type (S for square cross-section, and C for circular cross-section); the second letter refers to the type of reinforcement (G for GFRP, B for BFRP); the number after the second letter provides information about the width/diameter of the cross sectional area; the following letter denotes the tie material (S for steel, G for GFRP, and B for BFRP), followed by the eccentricity in mm; and, finally, the last number provides information about the concrete compressive strength used. As an example, S-G180-S80-40 indicates a square column of 180 mm width, reinforced with GFRP bars and steel ties, has a concrete compressive strength of 40 MPa, and loaded at 80 mm eccentricity.

The main objective of considering the 10 groups listed in Table 5 was to investigate the effect of the different parameters on the overall responses of the FRP-RC columns and their interaction diagrams. For example, the difference between Group 1 and Group 2 is only the type of ties material (steel vs. GFRP) and the difference between Group 1 and Group 3 was the dimension of the square cross-section (180 mm vs. 200 mm). Furthermore, the concrete compressive strength considered for Group 10 was 30 MPa while the concrete compressive strength for the other nine groups was 40 MPa. The 450 columns were numerically analyzed, and the results are reported and discussed.

**Figure 12:**
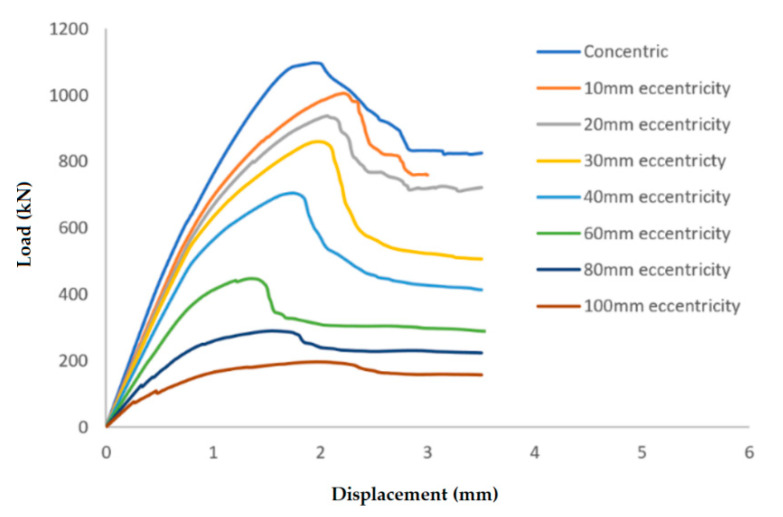
Load vs. displacement results for columns in Group 1, at the 1% reinforcement ratio.

Figure 12 presents a sample of the load vs. displacement curves for columns of the 1% reinforcement ratio in Group 1, predicted using the FE models.

**Figure 13:**
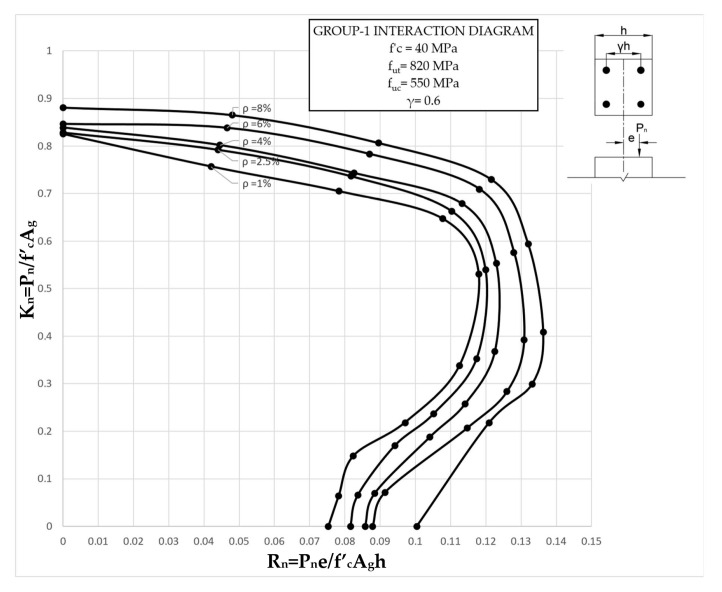
Group 1 interaction diagram.

**Figure 14:**
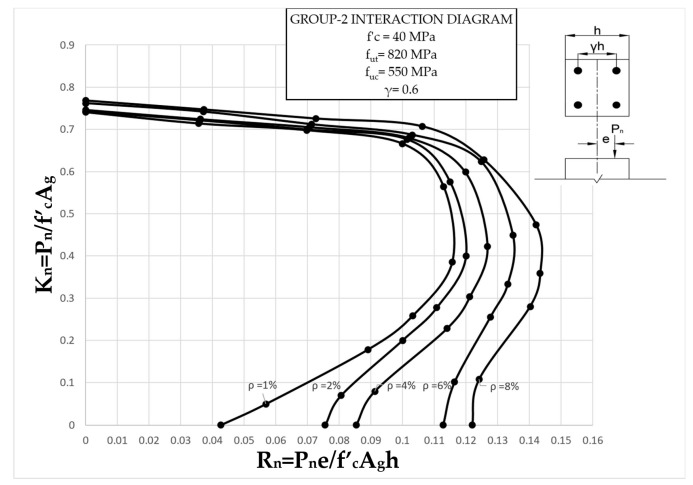
Group 2 interaction diagram.

**Figure 15:**
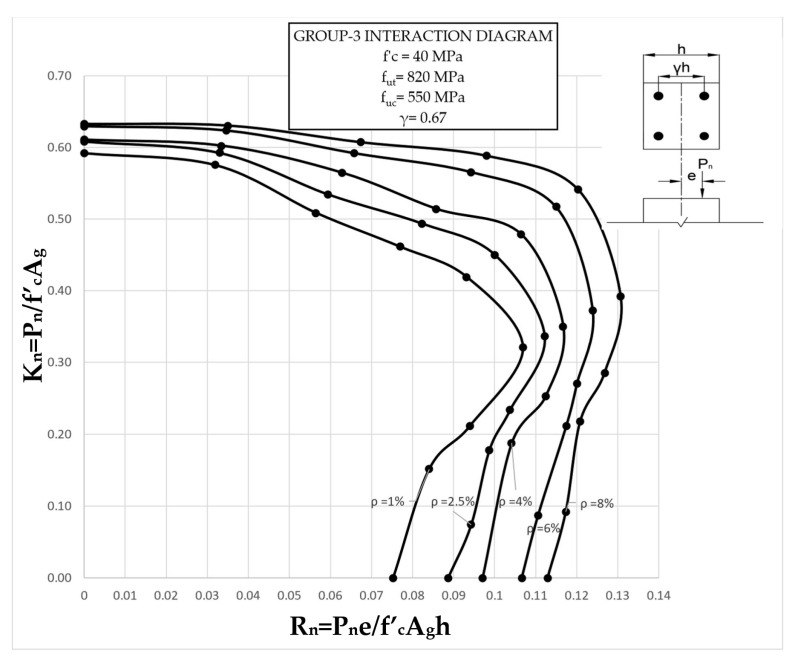
Group 3 interaction diagram.

**Figure 16:**
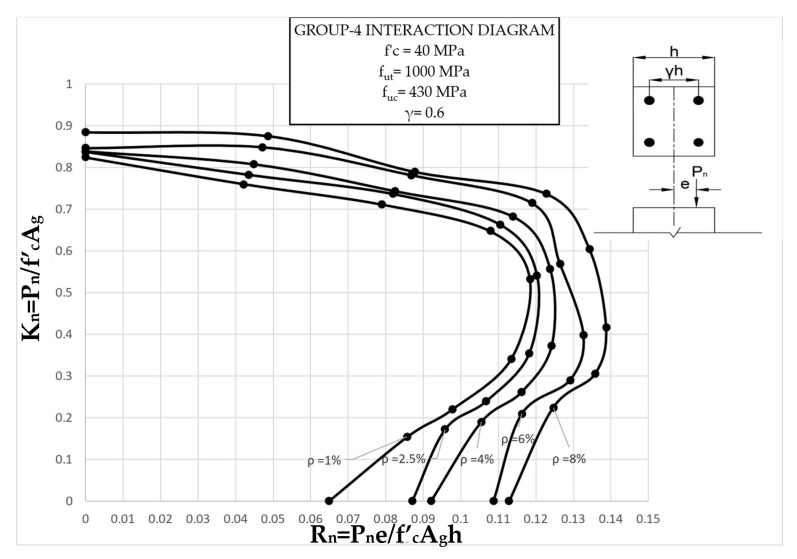
Group 4 interaction diagram.

**Figure 17:**
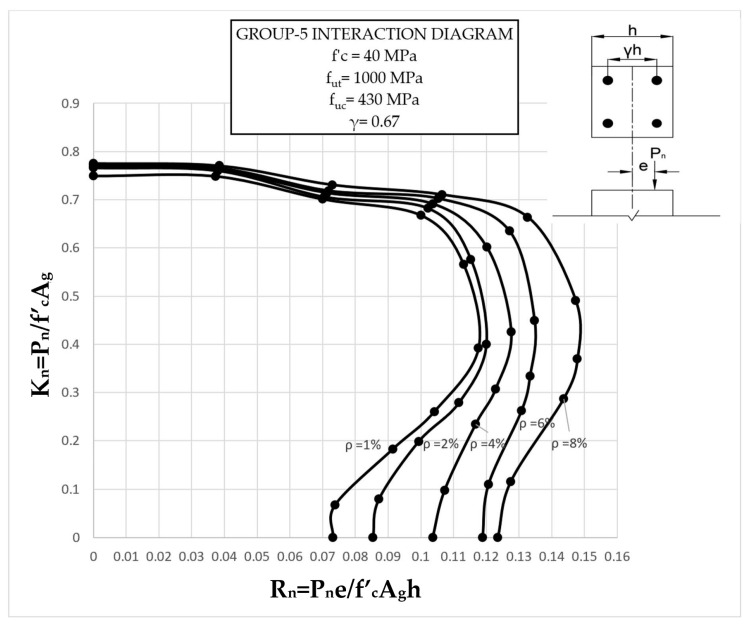
Group 5 interaction diagram.

**Figure 18:**
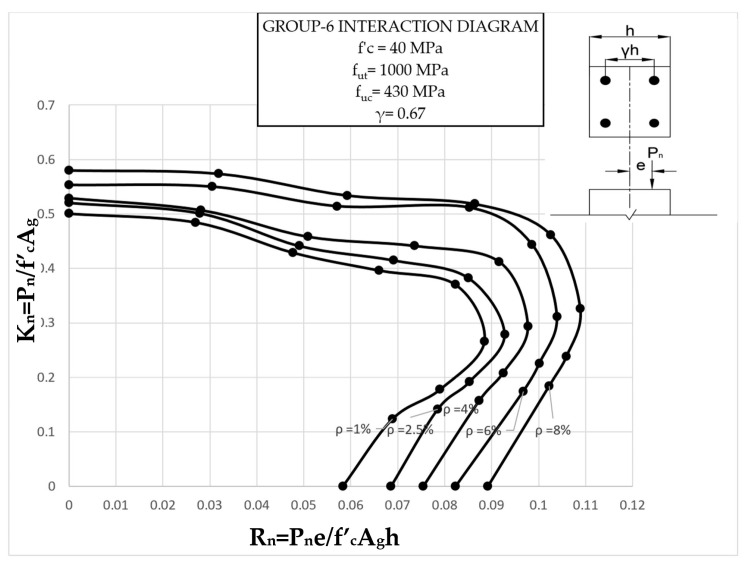
Group 6 interaction diagram.

**Figure 19:**
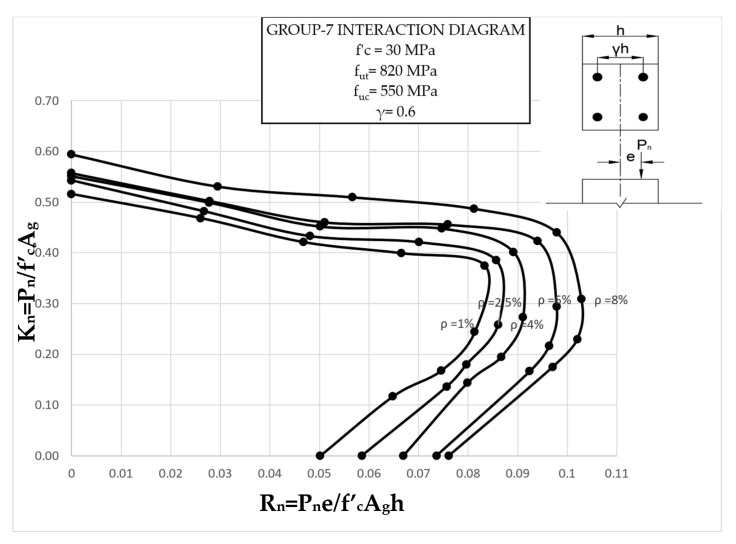
Group 7 interaction diagram.

**Figure 20:**
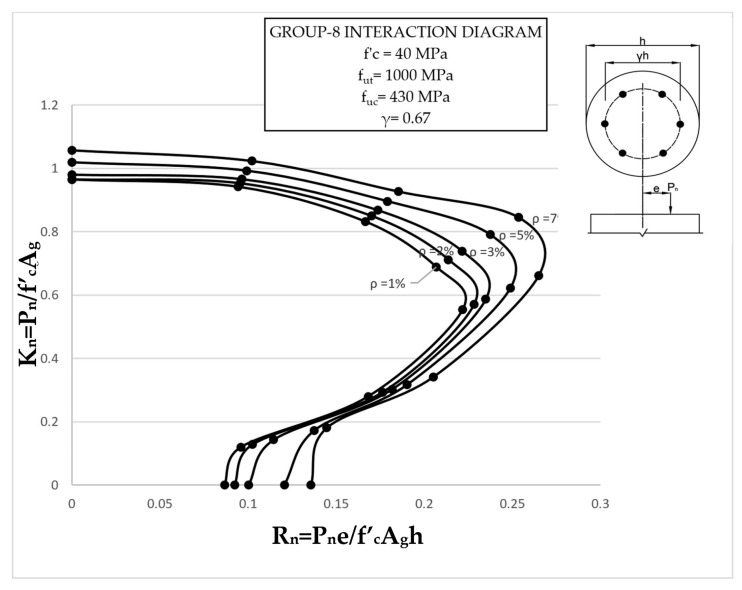
Group 8 interaction diagram.

**Figure 21:**
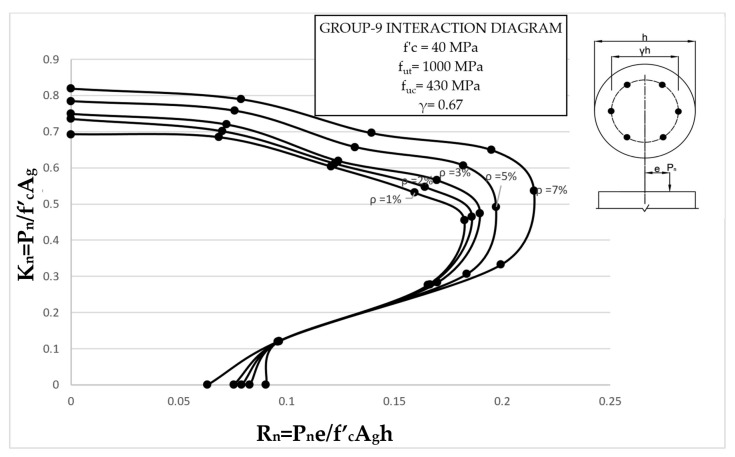
Group 9 interaction diagram.

**Figure 22:**
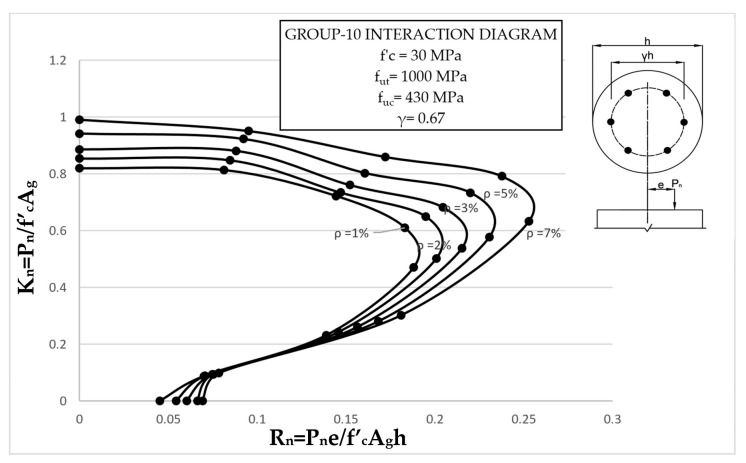
Group 10 interaction diagram.

The results clearly illustrate the transition in the stiffness of the columns as well as ultimate compressive loads over the different eccentricities considered. After obtaining the load vs. displacement results for all groups, interaction diagrams were developed for the five different reinforcement ratios, as shown in Figure 13, Figure 14, Figure 15, Figure 16, Figure 17, Figure 18, Figure 19, Figure 20, Figure 21 and Figure 22, which correspond to the columns in Groups 1–10, respectively.

The overall response and shape of these interaction diagrams were similar to the graphs presented in the ACI 318 [33] for steel-reinforced RC columns. Figure 13, Figure 14, Figure 15, Figure 16, Figure 17, Figure 18, Figure 19, Figure 20, Figure 21 and Figure 22 provide valuable information for the design of GFRP- and BFRP-RC columns.

The axial load vs. displacement results were also utilized to study the contribution of the GFRP and BFRP bars to the total compressive strength of the concentric FRP-RC columns in all groups. The confined concrete strength factor and ductility indices for the concentric FRP-RC columns were also calculated and compared. The confined concrete strength factor (f’_cc_) was calculated for each column as the difference between the peak load and force carried by the bars, divided by the confined concrete area (A_c_) delineated by the centerline of the ties ((P_max_−P_bar_)/A_c_). Values of the confined concrete strength factor (f’_cc_) for concentrically loaded columns are shown in Table 6.

Ductility is a desired property in structural design as it protects structures against unpredicted overloading and/or load reversals. It is therefore essential that RC columns possess adequate ductility. A method was developed by Pessiki and Peironi [34] in which the column ductility is calculated as the ratio of the ultimate axial displacement (*δu*) to the yield axial displacement (*δy*), given by *DI* = *δu*/*δy*. In this method, the yield displacement is estimated to be the axial displacement corresponding to the yield load or to the limit of the linear behavior. The ultimate displacement is assumed to be the axial displacement at 85% of the peak load in the post-peak descending portion of the load vs. displacement curve. The ductility index (DI) for the column is then calculated as the ratio of the displacements obtained for all columns, as shown in Table 6.

Additionally, values of the force carried by the concrete (P_concrete_), calculated as the difference between the ultimate load (P_max_) and the load carried by the bars (P_bar_), are shown in Table 6 for all the concentric columns.

## 5. Conclusions

The compressive strengths of small and large sizes of GFRP and BFRP bars were examined experimentally under static loads. The experimental results were then utilized as material input to develop nonlinear finite element (FE) models to simulate the axial behavior of concrete columns reinforced with these types of FRP bars. The FE model was initially validated with experimental results on FRP-RC columns and then used to conduct an extended parametric analysis to investigate the overall response of the FRP-RC columns. The parametric analysis included different column geometries and cross-sections, different reinforcement ratios, different ties materials, different concrete strengths, and different loading eccentricities. The main conclusions of this study are summarized below:

- The compressive strengths of both the GFRP and BFRP bars were much lower than their tensile strength values. Small size bars reported lower compressive strengths than large sizes.
- There were insignificant differences between columns reinforced with BFRP bars and columns reinforced with GFRP bars in terms of overall maximum load, displacement, and bar strength contributions.
- The contribution of GFRP and BFRP bars to the ultimate capacities of FRP-RC columns increases with the reinforcement ratio and was found to be around 5% for the lowest reinforcement ratio and up to 24% for higher reinforcement ratios.
- Increasing the longitudinal reinforcement ratio for the BFRP and GFRP columns did not significantly affect the ultimate capacities of the columns for concentric columns, but the increase was noticeable for eccentric-loaded columns.
- The ductility of columns reinforced with the BFRP or GFRP bars decreased when the reinforcement ratio increased. The ductility was higher when the circular cross sections were used.
- Using steel ties for confinement of RC columns resulted in higher capacities, higher concrete core confinements, and higher ductility than using BFRP and GFRP ties.

## Figures and Tables

**Table 1:** Tensile properties of the GFRP and BFRP bars [10,27].

Sample Material	Sample Diameter (mm)	Ultimate Tensile Stress (MPa)	Tensile Modulus of Elasticity (GPa)
GFRP	8	983.1 ± 32	
GFRP	12	976 ± 46	
GFRP	16	874 ± 39	44.9 ± 1.3
BFRP	8	1121.3 ± 56	
BFRP	12	1118.6 ± 31	
BFRP	16	1075.1 ± 37	49.3 ± 1.1

**Table 2:** Properties of the GFRP reinforcements.

Bar Diameter (mm)	Cross-Section Area (mm^2^)	Compressive Strength (MPa)	Average Strength (MPa)	Standard Deviation (MPa)
16.7	219	573	562.5	23
16.7	219	544.2		
16.7	219	551.5		
16.7	219	545.6		
16.7	219	598.1		
12.7	126.7	504.4	496.1	18
12.7	126.7	480.8		
12.7	126.7	510.7		
12.7	126.7	472.9		
12.7	126.7	511.5		
8	50.5	354.3	311.6	27.3
8	50.5	294.9		
8	50.5	287		
8	50.5	322.7		
8	50.5	298.9		

**Table 3:** Properties of the BFRP reinforcements.

Bar Diameter (mm)	Cross-Section Area (mm^2^)	Compressive Strength (MPa)	Average Strength (MPa)	Standard Deviation (MPa)
16.7	219	420	448.2	24.3
16.7	219	471.9		
16.7	219	440.8		
16.7	219	454.2		
16.7	219	441.8		
12.5	122.7	418	416.6	19.5
12.5	122.7	430.3		
12.5	122.7	390.3		
12.5	122.7	405		
12.5	122.7	439.2		
8.4	55.4	420.4	394.5	24.1
8.4	55.4	416.8		
8.4	55.4	362.7		
8.4	55.4	386.2		
8.4	55.4	386.2		

**Table 4:** Test matrix, specimens’ details, and results [32].

Column ID	Longitudinal Bars Type	Diameter (mm)	Reinforcement Ratio (%)	Load Eccentricity (mm)	Ties Type	Ties Spacing (mm)	Pmax (kN)
B16-40	BFRP	16	2.48	40	Steel	180	595
B20-40	BFRP	20	3.88	40	Steel	180	750
G16-0	GFRP	16	2.48	0	Steel	180	1050
S16-0	Steel	16	2.48	0	Steel	180	1300

**Table 5:** List of groups of RC columns considered in the FE parametric analysis.

Group	Column ID	Cross-Section	Longitudinal Reinforcement	Dimensions (mm)	Ties	*f*’_c_ (MPa)	Eccentricity (mm)
Group-1	S-G180-S **-40	Square	GFRP	180	Steel	40	0, 10, 20, 30, 40, 60, 80, 100 and Pure Moment
Group-2	S-G180-G **-40	Square	GFRP	180	GFRP	40	
Group-3	S-G200-S **-40	Square	GFRP	200	Steel	40	
Group-4	S-B180-S **-40	Square	BFRP	180	Steel	40	
Group-5	S-B200-S **-40	Square	BFRP	200	Steel	40	
Group-6	S-B200-B **-40	Square	BFRP	200	BFRP	40	
Group-7	S-G180-S **-30	Square	GFRP	180	GFRP	30	
Group-8	C-B200-S **-40	Circular	BFRP	203	Steel	40	
Group-9	C-B200-G **-40	Circular	BFRP	203	GFRP	40	
Group-10	C-B200-S **-30	Circular	BFRP	203	Steel	30	

Note: ** denotes 00, 10, 20, 30, 40, 60, 80, 100, and Pure Moment, which corresponds to the eccentricity.

**Table 6:** Confined concrete strength factors and ductility indices of all concentric columns.

Column ID	Reinforcement Ratio	P_max_	f’_cc_	DI	P_concrete_	P_concrete_/P_max_
S-G180-S00-40	1%	1096	52.5	1.56	1042	0.95
	2%	1100	51.5	1.66	1004	0.91
	4%	1114	50.7	1.41	965	0.87
	6%	1126	52.5	1.43	963	0.85
	8%	1170	54	1.56	964	0.82
S-G180-G00-40	1%	841	40.8	1.32	810	0.96
	2%	865	41.8	1.5	816	0.94
	4%	868	41.4	1.33	789	0.91
	6%	895	42.1	1.35	772	0.86
	8%	899	42.2	1.41	754	0.84
S-G200-S00-40	1%	1114	41.6	2.02	1080	0.97
	2%	1218	46	1.55	1157	0.95
	4%	1224	44.6	1.96	1109	0.91
	6%	1250	44.4	2.01	1064	0.85
	8%	1260	43.9	2.2	1018	0.81
S-B180-S00-40	1%	1095	53.2	1.56	1056	0.96
	2%	1113	53.5	1.43	1044	0.94
	4%	1114	50.7	1.63	965	0.87
	6%	1124	52.2	1.58	957	0.85
	8%	1175	54.1	1.68	966	0.82
S-B200-B00-40	1%	1228	45.3	1.33	1177	0.96
	2%	1256	47.5	1.34	1196	0.95
	4%	1258	46.3	1.3	1152	0.92
	6%	1265	45.1	1.39	1080	0.85
	8%	1271	44.4	1.43	1030	0.81
S-B200-S00-40	1%	811	40.2	2.13	785	0.97
	2%	843	41.7	1.46	812	0.96
	4%	857	42.2	1.93	803	0.94
	6%	896	43.6	2.02	800	0.89
	8%	940	44	2.13	786	0.84
S-G180-S00-30	1%	685.8	32.8	2.08	652	0.95
	2%	721	33.8	2.02	660	0.92
	4%	732	34.1	1.96	650	0.89
	6%	740	34.7	2.12	636	0.86
	8%	789	34.1	2.27	609	0.77
C-B200-S00-40	1%	1281	102.2	2.07	1267	0.99
	2%	1285	90.9	2.01	1109	0.86
	3%	1301	89	1.84	1063	0.82
	5%	1352	91.8	2.24	1041	0.77
	7%	1403	93.9	2.47	994	0.79
C-B200-G00-40	1%	920	64.4	2.1	799	0.87
	2%	976	65.8	2.2	802	0.82
	3%	995	66.4	2.24	794	0.8
	5%	1041	66.8	2.38	758	0.73
	7%	1087	71.5	2.45	756	0.7
C-B200-S00-30	1%	1087	81	2.17	1005	0.92
	2%	1133	81.9	2.25	999	0.88
	3%	1176	85.8	2.51	1025	0.87
	5%	1249	89.5	2.45	1015	0.81
	7%	1315	94.3	2.67	998	0.76
